# Progress in Medicine

**Published:** 1899-11-18

**Authors:** 


### Progress in Medicine.

### NEUROLOGY.

*(Continued from page 96.)*

**Insomnia.**—Bradbury,[5] in his Croonian lectures, has discussed at great length the nature and action of hypnotics, their subtle relations to each other, and the immense physiological differences which are caused by the substitution of one radicle for another. In his last lecture he came to the practical question of the treatment of insomnia, which he defines as a loss of the normal amount of sleep for the individual in question. What is sleeplessness in one person may not be so for another, for the amount of sleep varies with the individual, with age, with the mental constitution, and with the quality of the sleep. The causes of insomnia may be classed under four heads: (1) Irritative causes, (2) toxic causes, (3) psychical causes, and (4) causes arising from change in the mode of life. The class of irritative causes includes all forms of sleeplessness caused by pain, and milder irritations like uneasiness. A large number of diseases are accompanied by the presence of some poison in the blood, and such diseases are often attended by troublesome insomnia. Alcoholism, fevers, gout, Bright's disease, dyspepsia are some of these. Grief, shock, worry, mental anxiety, overwork, are most frequent causes of insomnia. Of causes arising from change in the mode of life, change of climate, especially to high altitudes or the seaside, late dinners to those unaccustomed to them may be mentioned. It is evident, from the multiplicity of these causes of sleeplessness, that no one line of treatment can be suitable for all cases. The first thing to do is to remove the cause. If it is, for instance, indigestion, acid or gouty dyspepsia, attention to the diet is essential. In cardiac disease and neurasthenia with low arterial tension, cardiac tonics are required, and so on. But often this method does not suffice. The removal of the cause does not bring back refreshing sleep. The cerebral cells have assumed an irritable condition, and it is necessary to bring them back to a normal state. It is here that hypnotics are of such great value. The best of these for any individual patient will vary according to his condition. There is no absolutely safe hypnotic, but experimental and clinical investigation shows that paraldehyde stands in the first rank. Chloralamide and chloralose are safer but slower than chloral hydrate. The sulphones are valuable, sulphonal being probably the best. But as a whole the bromides seem to be the least harmful, and in simple cases uncomplicated by other diseases, it is advisable to try them before resorting to any other drug. Sleeplessness from overwork, especially literary overwork, requires mental rest and change of air and scene. If the insomnia continues a glass of whisky and water at bed time, especially in those unaccustomed to its use, is often effectual. A mild hypnotic, such as bromide or sulphonal will often break the habit. Capsules containing 30 minims of turpentine given at bedtime are sometimes beneficial in the insomnia of overwork or worry. In nervous and hysterical women, and especially in women at the menopause, the bromides are very useful. The sleeplessness of the insane requires careful management. In the early stages of acute mania, bromides, chloral, hyoscine hydrobromide are useful; but a hot bath with the application of cold water to the head are most efficacious. In melancholia, with high arterial tension, paraldehyde or morphia is valuable, but one grain doses of erythrol tetranitrate, by reducing arterial tension, will often act better than anything else. In mild cases of delirium tremens sleep comes on after a time whatever treatment is adopted; in the more severe cases chloral and bromides are beneficial. Paraldehyde is recommended by some. Opiates may be given, but in most cases hyoscine is a more efficient remedy. Among the medical officers of the American army 20 grains of powdered capsicum in a bolus is the favourite hypnotic for this complaint. In pneumonia sleep usually comes at the crisis, but where this has not occurred a hypnotic such as chloralamide or paraldehyde will often act well. In pleurisy and other serous inflammations five or ten grains of Dover's powder may be used. In bronchitis chloralamide or chloral are safe, and as a rule opiates are to be avoided. The sleeplessness of asthma is relieved by remedies which cut short an attack, such as chloral, the fumes of Himrod's and other powders, hypodermic injection of morphia, and in some cases a five to ten grain dose of caffein. Paraldehyde both relieves the asthma and causes sleep. The insomnia of heart disease is benefited by digitalis and other heart tonics, but in some cases it is necessary to give morphia by the mouth, or, better still, hypodermically. Paraldehyde and chloralamide are also most useful. In chronic Bright's disease insomnia is occasionally very troublesome. Eliminants such as aperients should be tried; and if they do not succeed chloral should be given. The drug is safer in kidney than in heart disease, the reduction of blood pressure being usually beneficial. Erythrol tetranitrate often acts like a charm by reducing arterial pressure, even when sedatives have failed. When pain is the causal factor of insomnia morphia is usually the best remedy, and this should be pushed until relief is obtained. In cases of neuralgia, locomotor ataxy, and so forth some of the synthetic analgesics—antipyrin or phenacetin—are of value. Calcium chloride is a valuable remedy in insomnia due to pruritus.

**Agraphia.**—It has long been a matter of discussion whether or not a special centre existed in the cerebral cortex for the function of writing. Some authorities, *e.g.*, Elder, have maintained there is; others, *e.g.*, Bramwell, that there is not. The evidence has hitherto been inconclusive. But a case that would seem to decide the question in the affirmative has recently been recorded by Gordinier.[6] An intelligent woman, aged 37 years, began to suffer from severe frontal and occipital headache, with occasional attacks of vomiting. After three months there was impairment of vision, and numbness was complained of. There was not the slightest trace of aphasia, either motor or sensory. She was, however, totally unable to write, though she understood perfectly written language, and was able to read to herself or aloud. She could not write voluntarily, or form correctly a single letter, nor write from dictation or copy. She held the pen in a perfect manner, and performed the movements of writing, making, however, only a series of united curves. She was not ambidextrous, and could not write with the left hand. There was no indication of paralysis of any of the muscles of the right arm, and she had been able previously to write perfectly with the right hand. Double optic neuritis was present, more marked on the right than on the left side. The knee jerks were absent. Though there was no evidence of syphilitic infection potassium iodide and mercury were given for six weeks, but without any amelioration of the symptoms. In the interval cerebration had become quite slow. The patient then consented to operation. On incising the dura over the left Rolandic area, a quantity of subarachnoid fluid escaped with the formation of a hernia cerebri. The opening was enlarged forwards and careful search made for a lesion, but none was found. The condition of the patient becoming alarming, the wound was at once closed, drainage being provided for. The patient rapidly became worse, passed into a condition of unconsciousness, and died on the second day. On post-mortem examination a new growth was found occupying the foot of the second left frontal convolution, distinctly separated from the arm area by the precentral sulcus, which was well developed. Its longest cortical diameter was 2 cm., and over it the pia was adherent. On section the tumour was seen to extend downward and inward in the centrum semiovale as far as the root of the anterior horn of the lateral ventricle, and forward to near the apex of the frontal lobe. The third frontal convolution was perfectly normal, as was also its associated centrum ovale. On histological examination the tumour was found to be a glioma of rapid growth.

**Bulbar Paralysis.**—Schüle[7] describes a case of bulbar paralysis without anatomical changes. The patient was a woman, aged 53, in whom the bulbar symptoms commenced in May, and were well marked in June, 1897. The patient had a nasal tone of voice and difficulty in swallowing; fluids often came back by the nostrils. Weakness in the legs was noticed in August, and was followed by paresis of the arms. The pupils reacted normally, the deep reflexes were present, sphincters and sensation not affected, mental condition natural. There was a feeble but troublesome cough. On September 4th sudden violent dyspnœa occurred, ending in death in three hours. A necropsy was made, and the nervous system was examined macroscopically and microscopically with practically negative results. Jolly, Oppenheim, and others have described a kind of bulbar paralysis without anatomical changes, which has been termed "asthenic bulbar paralysis," because the symptoms were marked by increased fatigue. In Schüle's case this "fatigue symptom" was not present, and it shows that this symptom must not be over-estimated in classifying cases.

**Asthenic Bulbar Paralysis.**—Sinkler and Punton[8] have recently added additional cases to our scanty records of this disease. The essential feature of the disease is the rapid fatigue which was shown in the affected muscles when used, and the fact that there was not complete paralysis of any muscles, but rather a tendency to give out upon use. The intensity of the symptoms varied from time to time, and there were marked periods of remission. The disease began in the intrinsic ocular muscles, ptosis, with or without diplopia, being usually the first symptom. Then the muscles concerned in speech and deglutition were affected, and the lower extremities were sooner or later involved. The affection differed markedly from bulbar paralysis of organic origin, in that in asthenic bulbar paralysis there was never muscular atrophy, even in the tongue, nor were fibrillary twitchings observed. There was no complete paralysis of the muscles of the lips. In true bulbar paralysis there was seldom involvement of the third nerve, of the lower facial, or the motor branch of the fifth. Again, in none of the fifteen autopsies which had been made had any lesion been found in the nervous system.

**Locomotor Ataxy.**—Muskins[9] has investigated the retardation of the pain sense in locomotor ataxy. This retardation has for long been known, but its relations and localisation have been a matter but little inquired into. In one case of undoubted tabes the author found a circumscribed area of analgesia below the knee, while the sense for touch was intact. On careful examination, it was discovered that there was a zone of retardation of pain sense on the border of the analgesia area. Other patients, to the number of 31, were then investigated, with the result that the phenomenon was found to be constant. Zones of retardation of the pain sense are found where analgesia is developing, where the nerve fibres are slowly perishing. The zone of retardation varies much in broadness, from a millimetre or less to some centimetres.

[5] Bradbury, Lancet, June and July, 1899. [6] Gordinier, Amer. Jour. Med. Soc., Phila., May, 1899. [7] Schöle, Munch. Med. Woch., Mar. 29, 1899. [8] Sinkler and Punton, Med. Rec., July 22, 1899. [9] Muskins, Jour. of Nerv. and Med. Dis., July, 1899.

### DISEASES OF THE DIGESTIVE ORGANS.

(*Continued from page* 95.)

**Cirrhosis of the Liver** is the subject of a report by Vaughan Harley and Barratt,[22] who investigated the disputed production of interstitial hepatitis after ligature of one of the bile ducts under minute precautions. They find that complete obstruction of the duct does undoubtedly produce this affection, though sometimes other factors may vitiate the result, which accounts for varied conclusions of previous experimenters. Rolleston[23] lays down that cirrhosis in man is due to a toxic process which may be caused by many poisons, either absorbed direct from the intestines or reaching the liver by the hepatic artery. Generally these poisons enter by the portal vein, and alcohol is rather an antecedent condition than a direct agent. The possibility of cirrhosis being due to micro-organisms is supported by analogy, but is not finally proven. With regard to biliary cirrhosis, where we have a large, smooth liver and much jaundice, but no ascites, he thinks there are two pathological forms; (1) where the infection spreads up from the intestines along the larger bile ducts; and (2) when the inflammation starts in the smallest bile ducts and produces a descending cholangitis and pericholangitis. There is a form described by Hanot where bronzing of the skin and diabetes are also present. Osler[24] remarks that cases without diabetes have been seen, and describes two instances of his own, but confesses that we do not know its etiology. The appearance may suggest Addison's disease; the position of the pigment in the subcutaneous tissue, and in the cells of the sweat glands, as well as the reaction of iron which it gives, are important points in the diagnosis. The condition is probably due to blood destruction, and possibly to some kind of toxæmia. Chauffard,[25] in speaking of the enlargement of the spleen seen in some of these cases, advances the theory that the liver affection is sometimes secondary to that of the spleen. In malaria and leucæmia it would seem that the spleen is first attacked and that infection may pass through it to the liver, and it appears probable that in many of these forms of cirrhosis the liver may be affected by the blood from the splenic vein as much or more than from those of the intestines.

**Syphilis of the Liver** shows little difference between secondary and tertiary lesions. Adams[26] notices the remarkable frequency of liver troubles in congenital syphilis. Thus there are gummata, miliary gummata, a mixture of these and general fibrosis, and, finally, general atrophic cirrhosis. The fibroid changes are often due to the direct action of the toxins on the liver cells themselves, but with any of these forms there may be present skin affections of a secondary type. In the adult our knowledge of the affections during the secondary stage is very imperfect, but there are cases where the parenchyma is affected and jaundice results, probably from the irritation of the toxins and not from mere obstruction. Besides the forms seen in infants we find during late stages in adults old gummata undergoing involution, together with surrounding fibroid changes, or obsolete gummata without appreciable fibroid changes in the rest of the tissue. Thus, while the affections at both ages are anatomically identical, there is a greater amount of fibroid change in the infant and more of focal ones in the adult.

**Abscesses.**—Cantlie[27] divides these into suprahepatic or those in the broad ligament of the liver, intrahepatic, and infrahepatic. The first type is not due to dysentery, but to a lymphangitis following a chill. They tend to advance upwards through the diaphragm into the lung, and may discharge through a bronchus, leaving the liver quite healthy. Early aspiration is desirable, after Mansell's method. The amœba is not to be found in these abscesses. D. G. Marshall,[28] in speaking of the ordinary tropical abscess, admits that many of these follow non-amœbic dysentery, and are therefore free from the parasite, and in cases where they are really present they may be overlooked from several reasons. Thus they are rarely found in the fluid first drawn off, and they rapidly disintegrate and fall to pieces if not looked for at once, or kept at 80 deg. of heat, or fixed by perchloride.

**Gall Stones.**—Recent French observers, according to Brockbank,[29] have endeavoured to show that the cause of their formation is to be found in microbial action. Thus, Mignot argues from his experiments that they are produced when highly attenuated bacteria are present in a bladder which is possessed of little activity, though he confesses that in half the cases of long standing no organisms can be found. Hartmann points out that as calculi are permeable to fluids and to bacilli, any found in them may be late introductions, and not the cause of the formation. Brockbank adds that the increased formation of cholesterin occurs in catarrh of the mucous membrane, such as is seen in heart disease. Thus, in mitral stenosis he found gall stones in 19 out of 87 cases, and a bacillary infection can hardly be supposed to exist here. As to treatment, he suggests that gentle massage by the patient himself and exercises may aid by increasing the movement of the bile. Carr,[30] in speaking of treatment, advises morphia during the attack followed by a dose of calomel, and then salines for a month or six weeks. He would reduce the fats and sugar taken as food, forbid all indigestible articles; and as regards the various drugs which have been recommended, he finds their utility is based on the common power they have of increasing peristalsis. McGarland[31] thinks the production of calculis is due to many factors. Thus, they are more often found in middle life in persons of sedentary habits, heavy feeders, in women rather than in men, and rarely in tropical climates. Indeed, they often exist without symptoms, and occur in, perhaps, 7 per cent. of the whole population. He classifies calculi as (*a*) the common cholesterin, bilirubinate of calcium, (*b*) pure or laminated cholesterin, (*c*) bilirubin and bilirubinate of calcium, (*d*), and the rare calcium carbonate. Cholesterin is precipitated if the bile loses its alkalinity, or if its sodium salts are deficient. Possibly that found in calculi is derived from the epithelium of the bladder and not from the bile. The conditions for the formation of calculi are not known with certainty, but probably masses of inflammatory products and epithelium or bacteria form nuclei upon which bile salts precipitate. Cholesterin is deposited later, taking the place of organic substances as these are dissolved out. McWeeney[32] has recently shown a liver full of small abscesses, while the common duct was blocked by calculi, and the gall bladder was replaced by a carcinomatous growth. The abscesses swarmed with two varieties of the bacillus coli. He thought the bacilli were the cause of all, being followed by the calculi and finally by the new growth. A case of melanotic sarcoma of the liver, secondary to a growth in the eye, is discussed by H. D. Rolleston.[33] The organ was enlarged and nodular, melanin appeared in the urine, and similar growths followed in the skin. Many of these melanotic tumours approach the carcinomatous type. In fact, opinion is divided whether many of them are not true carcinoma. Jaundice was not present, and seems uncommon in these cases. The urine was clear but became dark on exposure, and gave a black ring on the addition of nitric acid or of perchloride of iron. The writer notices the importance of using both tests in similar cases, where a clear urine may cause the disease to be overlooked. An analogous danger is mentioned by Hayem in a paper on a variety of catarrhal jaundice. Here the urine may be quite free from bile pigment. The jaundice is persistent, and may be general or localised, the fæces are normal in colour, and the liver nearly normal in size. The patients suffer from hyperpepsia and neurasthenic symptoms.

**Yellow Atrophy.**—C. Douglas,[35] in reporting a case in a pregnant woman, refers to the variety of the disease, and mentions that leucin only was found in the urine, with some bile pigment. Indeed, both leucin and tyrosin are often absent, and when present only represent the failure of the liver to destroy useless material. He regards the disease as the result of some poison found in the bowels which destroys the liver, and that, as a result, the organism is poisoned by the products of its own metabolism. Three cases occurring in male coolies are reported from Fiji by H. Noble Joynt,[36] all of which proved fatal, though in one there was no jaundice, but general peritonitis and ascites. Kvjatkowsky[37] analyses the recorded instances of emphyæma of the liver, and concludes that the active cause is to be found in the bacillus ærogenes capsulatus, which differs from the B. coli in having a capsule, staining by Gram's method, being non-motile, and showing a special form of growth on potatos. It is often associated with pus cocci, and enters the organism by the veins and lymphatics.

**Intestinal Obstruction.**—The choice of nourishment which leaves no solid residue is sometimes important, especially in malignant disease. W. H. Dickinson[38] speaks highly of whey, clear beef tea, sugar, alcohol, and arrowroot, which are entirely soluble, and yet supply in combination the chief elements needed. Even in typhoid he gives sugar, arrowroot, and the common egg and brandy mixture in addition to milk and beef-tea. The instances in which the effects of plumbism have been mistaken for appendicitis are very instructive. T. P. Lord[39] mentions two cases where laparotomy was performed, one showing the ordinary signs of appendicitis, and the other being a supposed stricture of the colon. In the latter the colon was found spasmodically contracted to the size of a finger for 10 inches, and in the former a mass of small intestine was similarly affected. Another case of J. B. Murphy's is quoted where a piece of intestine was found to be like a solid cord three-eighths of an inch in diameter. After ten minutes' exposure to the air the spasm yielded and the bowel dilated. Oliver has seen similar conditions in animals poisoned by lead, but the demonstration of this condition in man is a new fact of great importance.

**Appendicitis.**—Rolleston[40] quotes a number of instances showing that traumatism may be the cause of appendicitis. In one there was a blow, in another a fall from a bicycle, and in a third taxis for the reduction of a hernia. Still found thread-worms in the appendix 25 times in 200 autopsies, and in one case there were 111 worms in that organ. Pain during life had been complained of in the right iliac region, and the appendix showed inflammatory changes of a catarrhal character. T. H. Caisteres[41] points out a possible explanation of the occasional curious recoveries of severe cases of appendicitis without operation. He gives drawings showing the actual position of the appendix in some cases of his own, where it lies flat on and adherent to the cæcum beneath the peritoneum. In such conditions perforation takes place more easily into the bowel, and the whole process may come to an easy and harmless termination.

[22] Brit. Med. Jour., July 22. [23] Quarterly Med. Jour., July. [24] Lancet, Aug. 12. [25] La Semame Med., May 24. [26] New York Med. Jour., April 22. [27] Lancet, Aug. 19. [28] Brit. Med. Jour., June 10. [29] Med. Chron., May. [30] New York Med. Jour., April 22. [31] Inter. Med. Mag., May. [32] Dublin Jour. Med. Sci., Aug. 1. [33] Lancet, May 13. [34] Lancet, June 3. [35] Glasgow Med. Jour., May. [35] Brit. Med. Jour., July 8. [37] Inter. Med. Mag., June. [38] Lancet, June 17. [39] Lancet, May 10. [40] Practitioner, Aug. [41] New York Med. Jour., Aug. 12.

